# The association of cognitive impairment with quality of life and functional impairment in Ugandan first-episode psychosis patients: a cross sectional study

**DOI:** 10.1186/s12955-022-02020-x

**Published:** 2022-07-23

**Authors:** Emmanuel K. Mwesiga, Andrew S. Ssemata, Joy Gumikiriza, Angel Nanteza, Anne Jacqueline Nakitende, Juliet Nakku, Dickens Akena, Noeline Nakasujja

**Affiliations:** 1 Department of Psychiatry, Makerere University, Kampala, Uganda; 2 Butabika National Referral Mental and Teaching Hospital, Kampala, Uganda

**Keywords:** Cognitive impairment, First-episode psychosis, Quality of life, Functional impairment, Low-income country, Uganda, WHOQOL-BREF, MINI

## Abstract

**Introduction:**

Cognitive impairment is common in first-episode psychosis patients and often associated with poor quality of life and functional impairment. However, most literature on this association is from high income countries and not low resource countries like Uganda. We aimed to determine the association between cognitive impairment with quality of life and functional impairment in Ugandan first-episode psychosis patients.

**Methods:**

At Butabika national psychiatric hospital of Uganda, we enrolled 94 first-episode psychosis patients aged 18–60 years with a confirmed first-episode of psychosis and no previous treatment with antipsychotic medication. Neuropsychological assessment was performed using the MATRICS consensus cognitive battery (MCCB). Quality of life and functional impairment were assessed using the brief version of the World Health Organisation Quality of Life scale (WHOQOL-BREF) and the MINI International Neuropsychiatric Inventory (MINI) respectively. Linear regression analyses determined the association between impairment in different cognitive domains with various quality of life and functional impairment domains while controlling for age, gender and level of education.

**Results:**

High scores in the reasoning and problem solving cognitive domain were associated with better quality of life in the psychological domain of WHOQOL-BREF (*p* = 0.029). For functional impairment, high cognitive scores in the domains of speed of processing (*p* = 0.018), reasoning and problem solving (*p* = 0.015), working memory (*p* = 0.017) and visual learning and memory (*p* = 0.002) were associated with psychosis “*having a greater impact on other members of the family*” on the MINI. Higher scores in the social cognition domain were associated with “*less aggressive and disruptive behaviour*” (*p* = 0.003).

**Conclusion:**

Cognitive impairment in Ugandan first-episode psychotic patients is associated with both poorer quality of life and functional impairment. Remediation of cognitive function may be a plausible intervention to improve outcomes in Ugandan first-episode psychosis patients.

## Introduction

The Global Burden of Disease ranks psychosis as the single most disabling medical condition in the world [1, 2]. Schizophrenia has a disability weight of 0.778 implying that patients are more than three quarters on their way to death, often by suicide and non-communicable diseases like hypertension, diabetes and malnutrition [3–6]. In 2016, 16% of the world’s population had mental disorders with more than 80% of these people living in the low and middle income countries (LMICs) [7, 8]. In sub-Saharan (SSA), psychosis accounts for 25–30% of disability due to mental disorders driven by a 100–200% increase in incident cases between 1990 and 2017 [9, 10]. In Uganda, psychotic disorders are the most prevalent and disabling conditions in patients presenting for the first time at the National Psychiatric hospital in Uganda [11, 12].

Cognitive impairment is a key domain of psychotic disorders and a greater driver of disease burden than positive, negative or affective symptoms [13]. Cognitive function in psychotic disorders is greatly impaired at the first-episode of psychosis (FEP) even before onset of antipsychotic medication [14]. The impairment is often in seven different domains of (1) working memory, (2) attention/vigilance, (3) verbal learning and memory, (4) visual learning and memory, (5) reasoning and problem-solving, (6) information processing speed, and (7) social cognition [15, 16]. Cognitive impairment in FEP patients has been associated with poor quality of life, worse long-term educational achievement and work functioning [17–24]. The various cognitive domains however do not equally impact functional outcomes in patients with psychotic disorders [23, 25]. For example, one systematic review that included seventeen different studies reported verbal memory and attention vigilance were more strongly associated with functional outcomes than other cognitive domains [26].

The literature on the association between cognitive impairment with both quality of life and functional impairment in FEP patients from LMICs is limited [27–29]. Comprehensive tests like the MATRICS consensus cognitive battery (MCCB) are rarely used in assessing for cognitive impairment in FEP patients from LMICs [30]. Objective measures like the brief version of the World Health Organization quality of life scale (WHOQOL-BREF) or disability assessment schedules of the Mini International Neuropsychiatric Interview (MINI) are also not frequently used to describe quality of life or functional impairment in this population [31–34]. Therefore, the association between cognitive impairment with quality of life and functional impairment is unknown. It is also unclear if some cognitive domains are associated with worse quality of life and functional impairment than others [33, 35].

### Study aim

We determined the association between cognitive impairment, measured by the MATRICS consensus cognitive battery; with both quality of life and functional impairment in Ugandan first-episode of psychosis patients. Determining which neurocognitive domains are associated with outcomes may help in the development of interventions like cognitive remediation that are already in use in high income countries (HICs) [15, 36, 37].

## Methods

### Study design and setting

This was a cross sectional study design undertaken from the Butabika National Psychiatric Mental referral hospital in Uganda. This 600-bed facility is in the central region of Uganda and serves both in-patients and outpatients. It has four acute admissions wards and two convalescent wards, in which patients with clinical resolution of symptoms are managed before discharge. The average bed occupancy rate is 149% and most patients presenting for the first time have a psychotic disorder [11]. As a national referral hospital, it receives patients from various regions of the country. The hospital also provides care for non-psychiatric illnesses through outpatient clinics for antenatal care, HIV/AIDS, dental treatment, and common infections like malaria.

### Sample size calculation for linear regression analyses

To determine the minimum sample size for linear regression analyses, a-priori sample size testing was performed. We used an a-priori Sample Size Calculator for Multiple Regression which calculates the minimum required sample size for a multiple regression study, given the desired probability level, the number of predictors in the model, the anticipated effect size, and the desired statistical power level. For an anticipated effect size of 0.15, desired probability level of 0.05 and desired statistical power of 80% we calculated minimum sample sizes of 103 participants for seven predictors in the model (seven cognitive domains) and 127 participants for twelve predictors in the model (twelve functional impairment questions). Therefore, a minimum sample size for regression analyses of 127 participants was used.

### Study participants

Adult in-patients on the four acute admission wards of Butabika hospital were recruited. Inclusion criteria were a confirmed psychotic disorder, being antipsychotic naïve, aged 18–60 years and able to give informed consent. A cut-off age of 18 years for the first episode of psychosis was applied to mitigate the challenges of neuropsychological assessment in adolescents versus adults. In Uganda, patients older than 60 years are deemed elderly and these individuals were excluded from participation to eliminate potential effects of normal ageing and dementia. Participants with prior substance use, HIV/AIDS, Syphillis and those with acute illnesses requiring urgent medical attention were excluded.

As is required in neuropsychological assessment, a sample of healthy peers matched for age, gender and education were also recruited from the outpatient dental department at Butabika Hospital. These participants are required to generate normative values for cognitive function in this population although they were not included in the final analysis. Neuropsychological assessment was performed if a particpant had (1) no evidence of psychosis or substance use, as assessed by the MINI; and (2) no evidence of HIV/AIDS or Syphilis. A complete detail of the healthy population has been described previously [38].

## Study instruments

### Sociodemographic questionnaire

Collected variables such as age, gender, the minimum level of education attained, diet, housing and employment status. Additional phenotypic data like ethnicity was also collected.

### MINI international neuropsychiatric inventory

The MINI version 7.0.2 includes a section that assesses for disability/functional impairment. The assessment of disability or functional impairment is based on the 2nd edition of the World Health Organization Disability Assessment Schedule (WHODAS 2.0). It consists of questions that assess “how much one’s symptoms have disrupted their ability to function in 12 areas of their lives.” These 12 areas include *work or school work, social life or leisure activities, family life and home responsibilities, ability to get along with people, ability to take care of oneself, made one disruptive or aggressive towards others, ability to manage money, ability to get around physically, spiritual or religious life, Condition having an impact on other members of the family* [39]. The responses are scored on a Likert scale that runs from 0 to 10. Scores of zero imply the symptom has had no disruption on that aspect of life, 1–3 imply mild disruption, 4–6 moderate disruption, 7–9 severe disruption and 10 extreme disruptions.

### WHOQOL-BREF

The WHOQOL-BREF instrument comprises 26 items which measure quality of life in four domains of physical health, psychological health, social relationships, and environment. Under each domain are various facets that are assessed for. Some of the facets included in the physical health domain include activities of daily living, work capacity, sleep and rest, mobility, energy and fatigue. The facets included in the psychological domain include self-esteem, bodily image and appearance, negative and positive feelings, and spirituality. Social relationships include personal relationships, social support and sexuality. Environment includes facets like financial resources, participation in leisure activities, security and transport. The WHOQOL-BREF has been used previously in settings such as ours and can be administered in approximately ten minutes [40–42]. The scores of the 26 questions are standardized from 0 to 100 with higher scores in a specific domain implying a better quality of life.

### MATRICS consensus cognitive battery (MCCB)

It is suggested as the gold standard the assessment of cognition in patients with psychosis. It assesses for impairment in all the seven key cognitive domains using ten different tests [16]. The Trail Making Test (TMT): Part A, Brief Assessment of Cognition in Schizophrenia (BACS): symbol coding, and Category Fluency: Animal Naming (Fluency) assesses for speed of processing. Hopkins Verbal Learning Test-Revised (HVLT-R) for verbal learning and memory. The Wechsler Memory Scale-Third Edition (WMS-III): Spatial Span and Letter-Number Span (LNS) assesses for working memory. Neuropsychological Assessment Battery (NAB): Mazes assesses for reasoning and problem solving. The Brief Visuospatial Memory Test-Revised (BVMT-R) assesses for visual learning and memory. Mayer-Salovey-Caruso Emotional Intelligence Test (MSCEIT): Managing Emotions (D & H) assesses for social cognition and the Continuous Performance Test- Identical Pairs (CPT-IP), MATRICS International Version 2 assesses for attention/vigilance) [16]. Composite scores are generated for each of the seven domain scores by summing the raw scores of individual tests per domain. These composite scores are then transformed to z-scores, using the means and standard deviations of the control group. Higher z-scores imply better cognitive function.

### Positive and Negative Syndrome Scale (PANSS)

This is a standardised 30-item clinician rated scale that assesses the presence and severity of positive and negative schizophrenia and bipolar psychotic symptoms [43]. It was used to ensure that patient psychotic symptoms had resolved prior to administering the MCCB, WHOQOL-BREF and MINI.

### Study procedure

At Butabika hospital, all in-patients with a diagnosis of psychosis were eligible for recruitment. A study nurse assessed these patients to determine further eligibility for enrolment. In a multi-stage process the study nurse reviewed patient files to confirm the patient was 18–60 years, had an admssion diagnosis for a psychotic disorder, was antipsychotic naïve and had no HIV/AIDS or syphillis. In this setting, all new admissions are screeened for HIV/AIDS and syphillis due to the greater prevalence in patients with severe mental illnesses [44]. This initial assessment was completed within 72 h of admission. Next, selected patients were followed up weekly using the PANSS till resolution of psychotic symptoms (scores of 2 or less on nine selected items of the PANSS [15–17]. On the convalesecnt ward, patients with symptom resolution had their psychosis diagnosis and no substance use confirmed using the MINI before enrollment into the study [45]. After obtaining informed consent, they were assesed for quality of life and functional impairement using the WHOQOL-BREF and the MINI. Thereafter, neuropsychological assessment using the MCCB was performed. Finally, chart abstraction of documented participants’ medication regimen and dosages.

### Data analysis

Data was analysed using Stata version 14 [46]. The outcome variables were the four domains of the WHOQOL-BREF and the 12 questions of the disability/functional impairment schedule of the MINI. The exposures were the seven cognitive domains on the MCCB. Sociodemographic characteristics were also classified as covariates. Descriptive statistics were used to determine the mean scores of the participants on the various tools. Pearson correlation coefficients were calculated between the scores of the seven different cognitive domains and WHOQOL-BREF domains. Linear regression analyses were performed to determine the association between impairment in specific cognitive domains and the four domains of quality of life, controlling for age and gender. Similarly, linear regression analyses were performed to determine the association between impairment in specific cognitive domains and the 12 variables of the MINI disability schedule. In all regression analyses, we controlled for age gender and level of education.

## Results

### Patient characteristics

94 participants with a first episode of psychosis were enrolled but only 90 participants had complete data collected which was entered into regression analyses. The median age of the sample was 26 years [IQR 21–33]. Most participants were female sex (75.5%), single (55.4%) and unemployed (51.1%). Additionally, most participants were presenting to the hospital for the first time (85.4%) and 12% of FEP patients had previously received antipsychotic medication for less than six weeks' duration. There was a wide variation in the duration of untreated psychosis with the mean time between the onset of symptoms and presenting to the hospital was 1 year [SD 3.05, range (0–18)]. Other participant characteristics are highlighted in Table 1.

**Table 1:** Baseline patient characteristics^a^

Factor	Level	N (%)
Age	Median (IQR)	26 (21; 33)
Age categories	18–19	13 (13.8)
	20–24	27 (28.7)
	25–29	17 (18.1)
	30–34	15 (16.0)
	35–39	12 (12.8)
	40–49	7 (7.5)
	50–59	3 (3.2)
Gender	Male	23 (24.5)
	Female	71 (75.5)
Participant’s years of education	Median (IQR)	4 (2; 8)
Father years of education	Median (IQR)	3 (2–6)
Mother years of education	Median (IQR)	2.5 (2–4)
Current living arrangements	Renting	16 (17.4)
	Owns house	16 (17.4)
	Living with family	53 (57.6)
	No housing, Living on street	7 (7.6)
Ethnicity	Bantu	70 (76.1)
	Nilotics	4 (4.4)
	Nilo-Hamites	4 (4.4)
	Sudanic	3 (3.3)
	Hamites	11 (11.9)
Who is main source of income in the household	Self	29 (31.5)
	Father	14 (15.2)
	Mother	18 (19.6)
	Relative/guardian	21 (22.8)
	Organization	10 (10.9)
Marital status	Single	51 (55.4)
	Married	26 (28.3)
	Divorced	15 (16.3)
Current employment history	Student	7 (7.6)
	Formal employment	7 (7.6)
	Non formal/ casual employment	31 (33.7)
	Unemployed	47 (51.1)
Handedness	Right	90 (95.7)
	Left	4 (4.3)

^a^2 participants missing some baseline data

### Cognitive function of the participants

The mean standardized z scores for the 7 different cognitive domains are highlighted in the Table 2. All mean participant scores were lower than healthy controls in all the seven cognitive domains. Mean standardized score performance was best in the social cognition domain [mean (SD): − 0.55 (1.72)] and worst in the reasoning and problem solving domain [mean(SD): − 1.94 (2.20)].

**Table 2:** Mean standardized scores for the 7 cognitive domains

Domain	Mean (SD)	Range
Attention/vigilance	− 1.74 (2.28)	− 10.83 to 2.25
Reasoning and problem solving	− 1.94 (2.20)	− 9.57 to 1.80
Speed of processing	− 1.24 (1.14)	− 6.33 to 0.63
Verbal learning and memory	− 0.89 (1.46)	− 4.24 to 1.77
Visual learning and memory	− 1.76 (2.06)	− 7.13 to 2.27
Working memory	− 1.13 (1.70)	− 5.42 to 2.83
Social cognition	− 0.55 (1.72)	− 5.81 to 3.53

### Association between quality of life and cognitive impairment

The mean (SD) scores of the quality of life domains were 55.5 (1.9), 59.1(2.2), 45.5(2.6) and 51.3(2.3) for the physical health, psychological, social relationships and environment domains respectively. Significant associations remain only for the association between the reasoning and problem solving cognitive domains with the psychological domain on the WHOQOL-BREF tool [β = 3.16(CI 0.34–5.97; *p *value = 0.029)]. Regression coefficients between the other cognitive and QOL domains are shown in the Table 3.

**Table 3:** Association between cognitive impairment and QOL (outcome)—adjusted for age, gender, education level

Factor	Physical health β (95%CI)	*P* value	Psychological β (95%CI)	*P* value	Social relationships β (95%CI)	*P* value	Environment β (95%CI)	*P* value
Speed of processing	1.57 (− 2.56; 5.70)	0.448	− 0.73 (− 5.96;4.51)	0.781	− 2.69 (− 8.71;3.32)	0.371	0.09 (− 5.00;5.18)	0.971
Attention	1.34 (− 1.30; 3.99)	0.311	1.39 (− 1.95;4.74)	0.404	− 0.69 (− 4.59;3.21)	0.724	0.92 (− 2.34;4.18)	0.572
Working memory	0.92 (− 2.17; 4.01)	0.550	− 0.27 (− 4.17;3.64)	0.891	− 0.86 (− 5.38;3.65)	0.702	2.07 (− 1.67;5.81)	0.271
Verbal learning	0.31 (− 3.05;3.67)	0.854	0.38 (− 3.85;4.62)	0.857	− 1.78 (− 6.65;3.10)	0.467	0.25 (− 3.86;4.37)	0.902
Visual learning	2.31 (− 0.04;4.67)	0.054	1.80 (− 1.25;4.85)	0.240	0.11 (− 3.49;3.71)	0.950	2.72 (− 0.17;5.62)	0.065
Reasoning and problem solving	1.23 (− 1.10;3.57)	0.293	**3.16 (0.34;5.97)**	**0.029**	1.49 (− 1.93;4.91)	0.385	2.45 (− 0.34;5.24)	0.084
Social	0.36 (− 2.35;3.06)	0.791	1.23 (− 2.16;4.62)	0.468	1.65 (− 2.26;5.58)	0.399	2.23 (− 1.01;5.47)	0.223

Bold values are statistically significant

### Association between functional impairment and cognitive impairment

For functional impairment, most participants (96.6%) reported that the symptoms had “disrupted their ability to function at work or at school.” The proportions of patients who reported how much the symptoms had caused impairment in the different questions on the MINI are shown in Table 4.

**Table 4:** Functional impairment symptoms in the study sample

Impact of symptoms on different areas of life	Level	N (%)	Median score (IQR)
Q1: Work or schoolwork	NP Present	3 (3.4) 86 (96.6)	5 (0.8)
Q2: Social life or leisure	NP Present	7 (7.8) 83 (92.2)	5 (0.8)
Q3: Family life or responsibilities	NP Present	7 (7.8) 83 (92.2)	5 (0.8)
Q4: Getting along with people	NP Present	6 (6.7) 84 (93.3)	4 (0.6)
Q5: Personal and social relationships	NP Present	7 (7.8) 83 (92.2)	4 (0.7)
Q6: Understand and communicate with others	NP Present	28 (31.1) 62 (68.9)	0 (0.4)
Q7: Take care of self	NP Present	30 (33.3) 60 (66.7)	0.5 (0.3)
Q8: Disruptive or aggressive towards others	NP Present	23 (25.6) 67 (74.4)	2 (0.4)
Q9: Financially	NP Present	23 (25.6) 67 (74.4)	2 (0.5)
Q10: Ability to get around	NP Present	15 (16.7) 75 (83.3)	3 (0.5)
Q11: Spiritual or religious life	NP Present	17 (18.9) 73 (81.1)	2 (0.5)
Q12: Condition having an impact on other members of the family	NP Present	11 (12.2) 79 (87.8)	4 (0.10)

NP, not present

4 Participants missing data on functional impairment

Only 2 out of 90 participants reported not present on all the 12 items of the MINI version 7.0.2 includes a section that assesses for disability/functional impairment. Four cognitive domains of speed of processing [β = 0.92, *p* = 0.018], working memory [β = 0.64, *p* = 0.017], visual learning and memory [β = 0.63, *p* = 0.002] and reasoning and problem solving [β = 0.45, *p* = 0.015], were associated with the functional impairment items of “*the condition having an impact on other members of the family.”* Visual learning and memory was associated with “*ability to take care of self*” [β = − 0.31, *p* = 0.038]. Social cognition was associated with ““*aggressive or disruptive behaviour*” [β = − 0.54, *p* = 0.003]. Other regression coefficients are shown in Table 5.

**Table 5:** Association between CI and FI (outcome)—adjusted for gender, education level

Factor	Q1 β(95%CI) *p* value	Q2 β(95%CI) *p* value	Q3 β(95%CI) *p* value	Q4 β(95%CI) *p* value	Q5 β(95%CI) *p* value	Q6 β(95%CI) *p* value	Q7 β(95%CI) *p* value	Q8 β(95%CI) *p* value	Q9 β(95%CI) *p* value	Q10 β(95%CI) *p* value	Q11 β(95%CI) *p* value	Q12 β(95%CI) *p* value
Speed of processing	0.05 (− 0.55;0.64) *P* = 0.875	0.06 (− 0.57;0.69) *P* = 0.842	0.05 (− 0.59;0.69) *P* = 0.868	0.11 (− 0.53;0.75) *P* = 0.736	0.09 (− 0.56;0.74) *P* = 0.787	− 0.42 (− 1.07;0.23) *P* = 0.199	− 0.55 (− 1.11;0.01) *P* = 0.056	− 0.48 (− 1.06;0.10) *P* = 0.104	− 0.08 (− 0.75;0.59) *P* = 0.813	− 0.15 (− 0.66;0.36) *P* = 0.560	0.26 (− 0.42;0.94) *P* = 0.454	**0.92 (0.16;1.68)** ***P* = 0.018**
Attention/vigilance	0.10 (− 0.17;0.37) *P* = 0.463	0.11 (− 0.19;0.40) *P* = 0.475	0.17 (− 0.13;0.47) *P* = 0.266	0.27 (− 0.02;0.57) *P* = 0.069	0.12 (− 0.18;0.43) *P* = 0.431	0.08 (− 0.23;0.39) *P* = 0.616	− 0.08 (− 0.34;0.19) *P* = 0.578	0.13 (− 0.14;0.41) *P* = 0.342	− 0.08 (− 0.39;0.24) *P* = 0.619	0.06 (− 0.18;0.30) *P* = 0.646	0.00 (− 0.32;0.32) *P* = 0.988	0.26 (− 0.10;0.62) *P* = 0.159
Working memory	0.12 (− 0.27;0.52) *P* = 0.535	0.12 (− 0.31;0.55) *P* = 0.576	0.09 (− 0.35;0.53) *P* = 0.674	0.22 (− 0.22;0.66) *P* = 0.314	0.06 (− 0.38;0.51) *P* = 0.781	0.09 (− 0.36;0.55) *P* = 0.678	− 0.07 (− 0.47;0.32) *P* = 0.713	− 0.04 (− 0.45;0.37) *P* = 0.843	− 0.22 (− 0.68;0.24) *P* = 0.343	− 0.21 (− 0.56;0.14) *P* = 0.242	0.23 (− 0.24;0.69) *P* = 0.340	**0.64** **(0.12;1.16)** ***P* = 0.017**
Verbal learning and memory	− 0.01 (− 0.49;0.46) *P* = 0.958	− 0.08 (− 0.57;0.41) *P* = 0.756	− 0.04 (− 0.54;0.46) *P* = 0.876	− 0.02 (− 0.52;0.49) *P* = 0.952	− 0.14 (− 0.64;0.37) *P* = 0.592	− 0.01 (− 0.52;0.51) *P* = 0.979	− 0.09 (− 0.54;0.36) *P* = 0.693	− 0.28 (− 0.74;0.18) *P* = 0.225	0.17 (− 0.35;0.69) *P* = 0.514	− 0.14 (− 0.54;0.26) *P* = 0.497	0.24 (− 0.29;0.77) *P* = 0.379	0.39 (− 0.22;1.00) *P* = 0.203
Visual learning and memory	0.14 (− 0.16;0.45) *P* = 0.360	0.14 (− 0.19;0.47) *P* = 0.387	0.12 (− 0.22;0.45) *P* = 0.491	0.25 (− 0.08;0.59) *P* = 0.132	0.21 (− 0.13;0.55) *P* = 0.219	− 0.17 (− 0.52;0.17) *P* = 0.316	− ** 0.31** **(**− **0.60;**− **0.02)** ***P* = 0.038**	0.10 (− 0.21;0.41) *P* = 0.513	− 0.08 (− 0.44;0.27) *P* = 0.631	0.00 (− 0.27;0.27) *P* = 0.988	0.28 (− 0.08;0.63) *P* = 0.122	**0.63** **(0.25;1.02)** ***P* = 0.002**
Reason and problem solving	− 0.02 (− 0.30;0.26) *P* = 0.891	0.05 (− 0.25;0.35) *P* = 0.720	0.08 (− 0.23;0.38) *P* = 0.617	0.18 (− 0.12;0.49) *P* = 0.232	0.08 (− 0.23;0.39) *P* = 0.598	− 0.17 (− .48;0.14) *P* = 0.278	− 0.07 (− 0.34;0.21) *P* = 0.627	0.27 (− 0.00;0.55) *P* = 0.053	− 0.12 (− 0.44;0.19) *P* = 0.444	0.11 (− 0.13;0.35) *P* = 0.377	0.15 (− 0.17;0.47) *P* = 0.356	**0.45** **(0.09;0.81)** ***P* = 0.015**
Social cognition	0.14 (− .23;0.51) *P* = 0.445	0.13 (− .26;0.53) *P* = 0.500	− 0.00 (− .40;0.40) *P* = 0.996	0.22 (− .18;0.62) *P* = 0.276	0.23 (− .18;0.63) *P* = 0.273	0.18 (− 0.23) *P* = 0.375	− 0.19 (− .55;0.17) *P* = 0.295	− ** 0.54** **(**− **.89;**− **.19)** ***P* = 0.003**	− 0.23 (− .65;0.19) *P* = 0.283	− 0.25 (− .57;0.07) *P* = 0.120	− 0.05 (− .48;0.38) *P* = 0.829	0.16 (− .34;0.65) *P* = 0.530

Bold values are statistically significant

Q1: Work or school; Q2: Social life or leisure; Q3: Family life or responsibilities; Q4: Getting along with people; Q5: Personal and social relationships; Q6: Understand and communicate with others; Q7: Take care of self; Q8: Disruptive or aggressive towards others; Q9: Financially; Q10: Ability to get around; Q11: Spiritual or religious life; Q12: Condition having an impact on other members of the family

## Discussion

We found that cognitive impairment was associated with both functional impairment and quality of life in Ugandan first episode psychosis patients. However different cognitive domains were associated with different patient outcomes.

### Association between impairment in different cognitive domains and functional impairment

The burden of functional impairment of 98% was higher than a study in Nigeria that found a prevalence of 78% [47]. Few studies in this setting have studied the association between functional impairment and cognitive impairment in low resource settings. The differences could be that the Nigerian study recruited outpatients that were chronically medicated and not first-episode psychosis patients. This supports the literature that shows worse functional impairment in chronic psychosis patients than FEP patients. This is important for policy makers in developing interventions to improve functioning early in the course of the illness [48].

Higher scores in the social cognition domain were associated with a decrease in “*disruptive and aggressive behaviour*”. This finding is in keeping with previous work from HICs were Computerized Social-Cognitive Training (CSCT) have shown to reduce aggression in patients with social cognitive deficits [49]. Currently, CSCT is not available in the Ugandan setting but trials on its effect in reducing disruptive behaviour and aggression may be beneficial [50].

Higher cognitive scores in the domains of speed of processing, working memory, visual learning and memory and reasoning and problem solving were associated with higher scores in “*the condition (FEP) having an impact on other members of the family*”. We hypothesize that even in FEP patients with high cognitive scores the other domains like delusions, hallucinations and disorganized thoughts and behaviours still make it difficult to care for these patients. This might highlight a need for family therapy in patients with high cognitive functioning [51–53].

In our study, poorer cognitive functioning in the visual learning and memory domain was associated with “*decreased ability to take care of oneself*”. To our knowledge this is the first study to highlight this association which has more significance for the Ugandan setting where in prior work we found the visual learning and memory domain most impaired in FEP patients [54]. Cognitive remediation therapies targeting this domain are still limited but further work is needed in the Ugandan context [55].

### Association between impairment in different cognitive domains and quality of life

Patients with FEP had significant associations between impairment in the reasoning and problem solving domain with the psychological domains of the WHOQOL-BREF. This finding is different from studies in HIC that have shown that the association between quality of life and impairment in the domains of social cognition [56–58], visual learning and memory [59], and verbal learning and memory [59]. Previous associations however in those cognitive domains were only present in patients with short durations of untreated psychosis and minimal psychopathology [60–62]. It is possible that the long durations of untreated psychosis and greater psychopathology in this setting impacted on the association between quality of life and cognitive impairment.

Most literature on the association between cognitive function and quality of life has been documented in elderly individuals [63, 64]. However, this study was performed in a relatively young population were the quality of life scores were found to be poor and associated with cognitive function. As these are still youthful individuals, it is imperative that interventions to improve quality of life are developed.

### Study limitations

The study used objective measures for quality of life, functional impairment and cognitive function which have not frequently been used in the literature of first-episode psychosis research. It was also performed in a low resource setting from which there is a dearth of literature on the association of cognitive function with quality of life and functional impairment. It would however have been better to use the longer version of the WHODAS 2.0 which categories functional impairment into 6 domains of understanding and communication, getting around, self-care, getting along with people, activities (household and school/work), and participation in society [39]. This brief version of the WHODAS does not categorise the functional impairment in this manner which may be easier for developing specific interventions for the different domains. Most studies that used the 12-item of the WHODAS 2.0 to assess for functional impairment found associations with a general cognitive impairment rather than impairment in specific cognitive domains [65, 66]. The few studies found associations in specific cognitive domains of verbal learning and memory as well as the reasoning and problem solving [67, 68]. However, most studies from high income countries (HIC) were performed in chronically ill patients and not first episode psychosis patients [14, 69, 70]. The participants were antipsychotic naïve first-episode psychosis patients with no prior substance use and these were excluded from the final dataset. However, substance use is a major driver of disability and is often comorbid in FEP patients [71–74]. Further studies including substance use history are required. Failure to obtain the prerequisite sample size of 127 patients is an acknowledged limitation. Finally, the study was conducted on a very specific patient group (first episode, antipsychotic naïve and resolved symptoms) that its generalizability to other patients with psychosis has limitations.

## Conclusion

Cognitive impairment, a common and disabling consequence in Ugandan first episode of psychosis patients is associated with worse quality of life and functional impairment. In this setting, there was only one association between impairment in the cognitive domain of reasoning and problem solving with the psychological domain of the WHOQOL-BREF. For functional impairment there were significant associations between the cognitive domains of speed of processing, working memory, visual learning and memory, reasoning and problem solving and visual learning and memory with the functional impairment items of “*ability to take care of self,*” “*aggressive or disruptive behaviour*” and “*the illness having an impact on the family.*” The domains associated with these outcomes differ from literature from high income countries. Therefore, further work is required to (1) understand the temporal relationships between cognitive impairment with quality of life and functional impairment through longitudinal studies and (2) the role of cognitive remediation programs in improving both quality of life and functional outcomes in this population.

## Data Availability

All data generated or analysed during this study is available from the corresponding author on request.

## Abbreviations

FEP: First-episode psychosis
WHOQOL-BREF: World Health Organisation quality of life inventory- brief version
MINI: MINI international neuropsychiatric inventory
